# Mitogenomes of Giant-Skipper Butterflies reveal an ancient split between deep and shallow root feeders

**DOI:** 10.12688/f1000research.10970.1

**Published:** 2017-03-06

**Authors:** Jing Zhang, Qian Cong, Xiao-Ling Fan, Rongjiang Wang, Min Wang, Nick V. Grishin

**Affiliations:** 1 Departments of Biophysics and Biochemistry, University of Texas Southwestern Medical Center, Dallas, TX, 75390-8816, USA; 2 Department of Entomology, South China Agricultural University, Guangzhou, Guangdong, 510640, China; 3 College of Life Sciences, Peking University, Beijing, 100871, China; 4 Howard Hughes Medical Institute, University of Texas Southwestern Medical Center, Dallas, TX, 75390-9050, USA

**Keywords:** phylogeny, mitochondria, sequence assembly, Hesperiidae, Megathymini

## Abstract

**Background:** Giant-Skipper butterflies from the genus *Megathymus* are North American endemics. These large and thick-bodied Skippers resemble moths and are unique in their life cycles. Grub-like at the later stages of development, caterpillars of these species feed and live inside yucca roots. Adults do not feed and are mostly local, not straying far from the patches of yucca plants.

**Methods:** Pieces of muscle were dissected from the thorax of specimens and genomic DNA was extracted (also from the abdomen of a specimen collected nearly 60 years ago). Paired-end libraries were prepared and sequenced for 150bp from both ends. The mitogenomes were assembled from the reads followed by a manual gap-closing procedure and a phylogenetic tree was constructed using a maximum likelihood method from an alignment of the mitogenomes.

**Results: **We determined mitogenome sequences of nominal subspecies of all five known species of *Megathymus* and *Agathymus mariae* to confidently root the phylogenetic tree. Pairwise sequence identity indicates the high similarity, ranging from 88-96% among coding regions for 13 proteins, 22 tRNAs and 2 rRNA, with a gene order typical for mitogenomes of Lepidoptera. Phylogenetic analysis confirms that Giant-Skippers (Megathymini) originate within the subfamily Hesperiinae and do not warrant a subfamily rank. Genus *Megathymus* is monophyletic and splits into two species groups. *M. streckeri* and *M. cofaqui* caterpillars feed deep in the main root system of yucca plants and deposit frass underground. *M. ursus*, *M. beulahae* and *M. yuccae* feed in the yucca caudex and roots near the ground, and deposit frass outside through a "tent" (a silk tube projecting from the center of yucca plant). *M. yuccae* and *M. beulahae* are sister species consistently with morphological similarities between them.

**Conclusions: **We constructed the first DNA-based phylogeny of the genus *Megathymus* from their mitogenomes. The phylogeny agrees with morphological considerations.

Giant-Skippers (Lepidoptera: Hesperiidae: Megathymini) are large, fat-bodied butterflies endemic to the North American continent ^1– 3^. Their caterpillars adapted to feeding inside large roots and fleshy leaves of *Yucca* and *Agave* plants and relatives. Protected from many predators living within their nutrition-rich food sources, Megathymini are larger in size than most other skippers, and don't feed as adults. Genus *Megathymus* is characterized by root-feeding caterpillars, mostly in *Yucca* plants, that build a "tent" (a silk tube projecting above the ground) at least prior to pupation. Caterpillars of the genus *Agathymus* live inside *Agave* leaves and make a "trap-door" (a round, hardened disk of silk) to close the entrance to their leaf chamber before pupation.

To better understand the evolution and phylogeny of *Megathymus*, we sequenced complete mitogenomes of all five known species from the genus: *M. yuccae*, *M. beulahae*, *M. ursus*, *M. streckeri,* and *M. cofaqui* ( http://www.butterfliesofamerica.com/L/Hesperiidae.htm). For most species, nominotypical subspecies from or near the type localities were used (see Figure 1 for specimen data; collected under the permit #08-02Rev). *M. beulahae* specimen, male paratype, was from the National Museum of Natural History collection (Smithsonian Institution, Washington, DC, USA). To confidently root the *Megathymus* tree, we also sequenced a complete mitogenome of *Agathymus mariae* as an outgroup. Methods for genomic DNA extraction, library construction, next-generation sequencing, and computational procedures followed those we reported previously ^4– 14^. The sequences have been deposited in GenBank and received accessions KY630500–KY630505.

**Figure 1.**
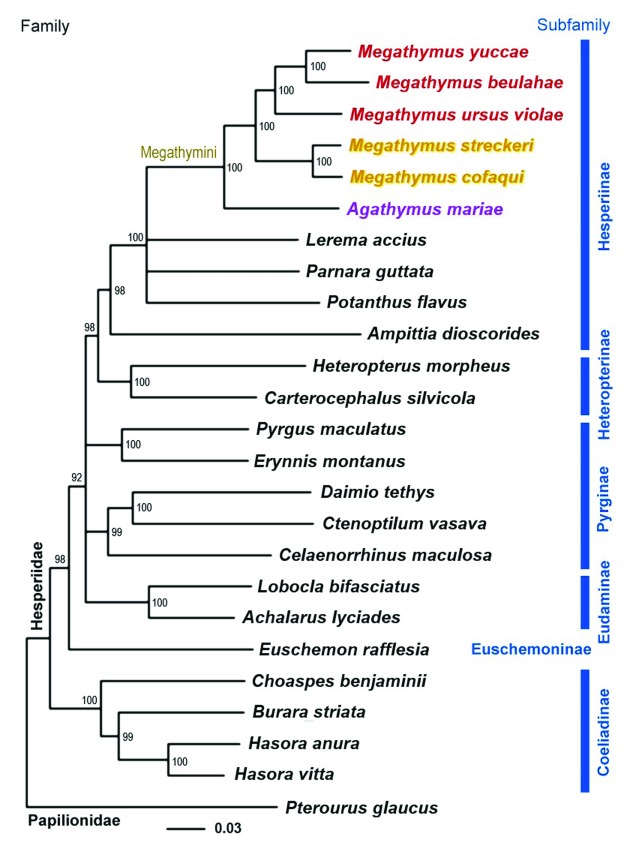
Maximum likelihood tree of complete mitogenomes of 24 Hesperiidae species rooted with *Pterourus glaucus* (Papilionidae). Species names for mitogenome reported here are colored red. Numbers by the nodes show bootstrap support values and branches; bootstraps less than 70% are collapsed. GenBank accessions for sequences and data for specimens with mitogenomes reported here are: *Achalarus lyciades* NC_030602.1; *Agathymus mariae mariae* KY630504, voucher NVG-1647, female, USA: New Mexico, Eddy County, 22-Sep-2013; *Ampittia dioscorides* KM102732.1; *Burara striata* KY524446; *Carterocephalus silvicola* NC_024646.1; *Celaenorrhinus maculosa* NC_022853.1; *Choaspes benjaminii* NC_024647.1; *Ctenoptilum vasava* NC_016704.1; *Daimio tethys* NC_024648.1; *Euschemon rafflesia* KY513288; *Erynnis montanus* NC_021427.1; *Hasora anura* NC_027263.1; *Hasora vitta* NC_027170.1; *Heteropterus morpheus* NC_028506.1; *Lerema accius* NC_029826.1; *Lobocla bifasciatus* NC_024649.1; *Megathymus beulahae beulahae* KY630505, voucher 11-BOA-13385G05, paratype, male, Mexico, Hidalgo, near Ixmiquilpan, highway 85, klm. 176, 19-Aug-1957; *Megathymus cofaqui cofaqui* KY630503, voucher NVG-1536, female, USA: Georgia, Burke County, 2-Aug-2013; *Megathymus streckeri streckeri* KY630501, voucher NVG-1461, male, USA: Arizona, Apache County, southeast of Holbrook, 19-May-2013; *Megathymus ursus violae* KY630502, voucher NVG-1504, male, USA: Texas, Pecos County, Glass Mountains, 7-Jun-2013; *Megathymus yuccae yuccae* KY630500, voucher NVG-1185, male, USA: South Carolina, Aiken County, 25-Feb-2013; *Papilio glaucus* NC_027252; *Parnara guttata* NC_029136.1; *Potanthus flavus* NC_024650.1; *Pyrgus maculatus* NC_030192.1.

All specimens, but one, were collected in 2013 and pieces of their muscles cut out of the thorax were preserved in 100% ethanol to ensure best DNA quality. However, *M. beulahae* paratype specimen was collected in 1957 ^15^ and stored pinned, spread and dry in a museum drawer for 60 years. DNA was extracted from its abdomen prior to genitalia dissection and produced good quality genomic reads resulting in a complete mitogenome assembly. Similarly to the results reported previously ^16^, we see that dry insect collections are an invaluable source of specimens for DNA studies; DNA can be extracted from Lepidoptera without damaging specimens beyond standard genitalia dissection procedure; and good quality DNA sequences can be obtained from specimens collected many decades ago.

Sequence comparison revealed that mitogenomes of all six species of Megathymini were very similar, about 15K base pairs in length, coding for 13 proteins, 22 transfer RNAs and 2 ribosomal RNA with gene order typical for mitogenomes of Lepidoptera. The A+T-rich control region is most variable in sequence and length and contains several direct repeats of about 360 bp present in all six species. Among Hesperiidae with available mitogenomes ^6, 10, 11, 13, 14, 17– 24^ these repeats are unique to Megathymini. The repeats cause difficulty with mitogenome assembly and their number remains uncertain.

To obtain the first DNA-based phylogeny of *Megathymus*, we constructed RAxML ^25^ (version 8.2.8, model GTRGAMMA, 100 bootstrap replicates) maximum likelihood tree from available high-quality mitogenomes of Hesperiidae ^6, 10, 11, 13, 14, 17– 24^ rooted with *Pterourus glaucus* (Papilionidae) sequence ^10^ ( Figure 1). While not giving confident resolution to the relationships between subfamilies Eudaminae and Pyrginae, the tree confirms the placement of Megathymini within the subfamily Hesperiinae ^26– 28^ and argues against historical treatment of Giant-Skippers at subfamily level. The tree resolves the Megathymini phylogeny with 100% bootstrap, supports monophyly of the genus *Megathymus* and suggests a split between the two species groups. The first group is formed by *M. streckeri* and *M. cofaqui*. Caterpillars of these species feed deep in the main root system of yucca plants and deposit frass underground ^2, 3^. They build a tent only prior to pupation and the tent usually projects from the ground surface. Males of these two species possess hair-like scales, particularly prominent on dorsal hindwing. *M. streckeri* and *M. cofaqui* are the closest sister species among *Megathymus* ( Figure 1). Due to their apparently close relationship and allopatric distribution, Scott has suggested that *M. streckeri* and *M. cofaqui* may be subspecies of the same biological species ^2^. However, the COI barcode sequences we obtained show about 4% divergence between them, revealing significant differences and supporting the two taxa as distinct species. COI barcode divergence in different populations of the same species mostly falls within 2% ^29^.

The second species group consists of *M. yuccae*, *M. beulahae* and *M. ursus*. Caterpillars of these species feed in *Yucca* caudex and in roots close to the ground, maintaining the tent throughout development and depositing frass outside the tent ^2, 3^. Males of these three species lack hair-like scales. *M. yuccae* and *M. beulahae* are sister species, as expected from their close morphological similarities. However, their COI barcodes show a very large divergence of 9%. This pronounced divergence was unexpected, because the two species are quite similar in appearance and some males are difficult to tell apart ( http://www.butterfliesofamerica.com/L/t/Megathymus_a.htm). The most noticeable difference between *M. yuccae* and *M. beulahae* is the larger white ventral hindwing spots in the latter species, frequently fused to form a bad, especially in females. However, these spots may be significantly reduced in males, frequently in the northern populations. Interestingly, *M. beulahae* is the only *Megathymus* species that feeds in yucca-like *Agave* plant ^1, 15^, but it is a confident sister of *Yucca*-feeding *M. yuccae*. *M. ursus* is a sister to these two *Megathymus*. *M. ursus* has rather different wing shape and patterns. The wings are narrower with more extended apex, forewing spots well-separated in *M. yuccae* form a band-like arrangement, and hindwings lack spots that females *M. yuccae* and *M. beulahae* possess.

In conclusion, we sequenced mitochondrial genomes of all five known species of *Megathymus* and one species of *Agathymus* as an outgroup, and constructed the first DNA-based phylogeny of *Megathymus*. The phylogeny is fully consistent with morphological and behavioral similarities between species. Our results support phylogenetic placement of Megathymini within the subfamily Hesperiinae and clarify the relationships between *Megathymus* species. In particular, the major phylogenetic split is between the shallow and deep yucca root feeders, and significant mitochondrial DNA divergences between *M. yuccae* and *M. beulahae* and between *M. streckeri* and *M. cofaqui* support the species status of these allopatric and similar in appearance taxa.
