# Supplementary material for: Mitogenomes of Giant-Skipper Butterflies reveal an ancient split between deep and shallow root feeders
Source: F1000Res. 2017 Mar 6;6:222. [Version 1] doi: 10.12688/f1000research.10970.1 (PMC5373422; doi:10.12688/f1000research.10970.1)
Supplement: Peer review history

# Peer review: Referee response for version 1

**DOI:** 10.5256/f1000research.11826.r21272

**Published:** 2017-03-27

**Authors:** Jiasheng Hao

**Affiliations:** 1 College of life sciences, Anhui Normal University, Wuhu, China

1. This work determined mitogenome sequences of nominal subspecies of all five known species of Megathymus and Agathymus mariae and constructed the phylogenetic trees of the main Hesperiinae groups, the results confirms that  Giant-Skippers (Megathymini) should not warrant a subfamily rank and that the monophyletic Megathymus  splits into two species groups. In addition, the reconstructed mitogenomic phylogeny is fully consistent with morphological and behavioral similarities between the closely related species of the genus Megathymus. Overall this interesting work deserves to be published and indexed.
2. The manuscript’s writing is good with an appropriate title and other content; the design, method and analysis is generally correct though some phylogenetic analyses should be more robust.
3. If the work considers the molecular dating of the Megatheymus divergence by molecular clock method, and incorporates the analysis of relevant earth environmental factors, the results and significance will be more remarkable.

I have read this submission. I believe that I have an appropriate level of expertise to confirm that it is of an acceptable scientific standard.

# Peer review: Referee response for version 1

**DOI:** 10.5256/f1000research.11826.r21230

**Published:** 2017-03-23

**Authors:** John A. Shuey

**Affiliations:** 1 The Nature Conservancy, Indianapolis, IN, USA

This paper is an interesting contribution to our understanding of the evolution of Megathymini and the phylogentic placement of the tribe within the Hesperiidae.   Using mitogenomes of all recognized species of Megathymus, the authors confirm morphologically-based  species and species groups.  Moreover – they identify a deep mitogenomic divergence between lineages that correspond with important life history traits that define species groups.

I have read this submission. I believe that I have an appropriate level of expertise to confirm that it is of an acceptable scientific standard.
